# Incidence density of hyperuricemia and association between metabolism-related predisposing risk factors and serum urate in Chinese adults: a cohort study

**DOI:** 10.3389/fendo.2023.1253470

**Published:** 2023-12-07

**Authors:** Hailun Liang, Jijuan Zhang, Hancheng Yu, Lijie Ding, Feng Liu, Jun Wang

**Affiliations:** ^1^ School of Public Administration and Policy, Renmin University of China, Beijing, China; ^2^ Institute for Medical Dataology, Cheeloo College of Medicine, Shandong University, Jinan, China; ^3^ Department of Health Management, Beijing Physical Examination Center, Beijing, China

**Keywords:** incidence density, hyperuricemia, serum urate, metabolism-related risk factor, cohort study

## Abstract

**Background:**

Evidence regarding the association between metabolism-related indicators and serum urate (SU) is limited. We aimed to obtain the incidence density of hyperuricemia and to explore the association between metabolism-related predisposing risk factors and SU.

**Methods:**

A total of 48,979 Chinese adults from the Beijing Physical Examination Center were included in the study. The partial least squares path model was used to explore the relationship between SU and metabolism-related risk factors. The generalized additive model was used for smooth curve fitting, showing the sex-specific associations of SU at follow-up with baseline fasting blood glucose (FBG) concentrations and age.

**Results:**

The incidence density of hyperuricemia was 78/1000 person-years. Baseline SU, age, sex, obesity, FBG, and lipid metabolism were significantly associated with SU at follow-up (all *P* values <0.05). Non-linear relationships were found between the baseline FBG concentrations and SU at follow-up, while U-shaped associations were observed between baseline age and SU at follow-up.

**Conclusions:**

The SU concentration is associated with several metabolism-related risk factors such as obesity and FBG. Recognition of these associations will aid in a deeper understanding of the multifaceted nature of SU regulation.

## Background

Serum urate (SU) is the final metabolite of purine compounds in the human body. Hyperuricemia occurs when the body’s purine metabolism and urate excretion are in disorder. Studies have reported a hyperuricemia prevalence of 21.4% among adults in the United States (1). In Europe, a cohort study in Ireland noted an increase in hyperuricemia prevalence from 20.1% to 24.5% between 2006 and 2014 (2), while in Italy, the prevalence is 6.3% among the general population (3) and 14.5% among individuals with hypertension (4). Also, Eastern Europeans with hypertension have a hyperuricemia prevalence of 25% (5). In Asia, Thailand’s prevalence stands at 10.6% (6). In mainland China, the pooled prevalence of hyperuricemia was 13.3% (7). Thus, hyperuricemia constitutes a growing threat to the public health.

The metabolic factors might be associated with the pathogenesis of hyperuricemia. Some studies have employed multivariable regression models to explore the associations of SU with fasting blood glucose (FBG), blood pressure (BP) or body mass index (BMI) (8–10). However, most of these studies were cross-sectional studies. Hence, the prospective association between SU and diverse metabolic factors remains unclear.

In this cohort study, we aimed to investigate the relationship between baseline metabolic risk factors and SU at follow-up in a large sample of Chinese adults using a relatively novel method named “partial least squares path model” (PLSPM). PLSPM is a structural equation model with few restrictions on the distribution of variables. PLSPM can be used to illustrate the importance of various factors that affect SU as well as reveal the interrelationships of various factors, and relevant findings may elucidate the underlying mechanisms behind the indicators.

## Methods

### Population

The Han Chinese adults aged from 18 to 94 years old who received annual medical examinations at Beijing Physical Examination Center joined this study. Beijing Physical Examination Center provided Beijing citizens with medical services widely, and the cohort established based on the agency could represent citizens in Beijing. At the beginning of the study, participants with incomplete information, suffering from hyperuricemia, cancer, hepatosis and renal dysfunction were excluded, and then the participants who met the inclusion criteria were followed up for hyperlipidemia. The subjects took the first medical examination in 2014, 2015 or 2016 respectively, and had at least two examination records during the follow-up. The interval between the first and the second physical examination was required to be at least 3 months. A total of 48,979 participants (24,273 men, 24,706 women) were included in the study. Flow chart of the study was shown in **Supplementary Figure S1**.

### Data collection and measurements

The age and sex of the subjects were registered in the information system. Under the condition of wearing light clothes and taking off shoes, the height and weight were measured with an electronic instrument. BMI was calculated as weight (kg) divided by the square of height (m^2^). A non-elastic, adjustable tape was used to measure the waist circumference (WC). The subjects rested for 5 minutes before measuring the systolic blood pressure (SBP) and diastolic blood pressure (DBP), and an electronic monitor was used to measure the blood pressure. After fasting for at least 12 hours, the blood samples of the subjects were taken away. Biochemical indicators such as FBG, total triglycerides (TG), total cholesterol (TC), high-density lipoprotein cholesterol (HDL-C), low-density lipoprotein cholesterol (LDL-C) and SU were tested using standard methods.

### Definition of hyperuricemia

Hyperuricemia was defined as the SU concentration >420 μmol/L (men) or >360 μmol/L (women) (11). We also performed a sensitivity analysis using cut-offs reported in the URRAH (Uric Acid Right for Heart Health) Project as requested by a reviewer (12).

### Statistical analyses

Continuous variables conformed to the normal distribution were expressed as mean ± standard deviation, and those not normally distributed were represented by the median and interquartile range (IQR). Student’s t test was used for comparison of continuous variables with normal distribution, and nonparametric Wilcoxon test was used for continuous variables not normally distributed. The *χ^2^* test was used for comparison of categorical variables between groups. The incidence density was calculated using the following formula:


$$\text{Incidence density=}\frac{\text{Number of new cases in a given time-period}}{\text{Total person-time at risk during that time-period}}$$

The PLSPM was used to explore the relationship between SU and metabolism-related risk factors. The PLSPM model is a kind of structural equation model, which consists of measurement models and structural models. The measurement model reflects the relationship between the observed variables and the latent variables, and the structural model is used to analyze the structural relationship between the latent variables (13, 14). In this study, latent variables included SU at follow-up, age, sex, obesity, FBG, lipid metabolism, blood pressure and baseline SU. Observed variables included SU at follow-up, age, sex, FBG, BMI, WC, TG, TC, HDL-C, LDL-C, SBP, DBP and baseline SU. Apart from SU at follow-up, which was measured at the end of the follow-up, the other observed variables were measured at baseline. Referring to relevant medical knowledge and literature, we established the measurement model, showing the relationship between latent variables and observed variables. The structural model was used to analyze the relationships between SU at follow-up and other latent variables. Considering that the follow-up time of different individuals was not necessarily the same, and the PLSPM couldn’t deal with the factor of follow-up time, therefore, we added a subgroup analysis to divide the population into different groups according to the follow-up time, and models of different groups were established and compared. The generalized additive model was used for smooth curve fitting, showing the sex-specific associations of SU at follow-up with baseline FBG concentrations and age. All statistical tests were two-sided tests, and a *P* value<0.05 was considered statistically significant. R 4.0.3 software was used for statistical analysis.

## Results

### Incidence density of hyperuricemia and participant characteristics

The total incidence density was 78/1000 person-years (115/1000 person-years for men, 42/1000 person-years for women) (**Supplementary Table S1**). Prevalence of hyperuricemia was shown in **Supplementary Table S2**.

**Table 1** showed the characteristics of the subjects of different sex grouped by hyperuricemia (hyperuricemia and non-hyperuricemia).

**Table 1:** Characteristics between men and women grouped by hyperuricemia^*^.

Characteristics	Men (n=24273)			Women (n=24706)		
	Hyperuricemia (n=4259)	Non-hyperuricemia (n=20014)	*P* value	Hyperuricemia (n=1560)	Non-hyperuricemia (n=23146)	*P* value
Age (years)	41.9 ± 14.1	43.9 ± 13.8	<0.001	46.3 ± 15.6	40.8 ± 12.8	<0.001
Baseline SU (μmol/L)	374.2 ± 34.6	321.5 ± 52.9	<0.001	304.4 ± 37.2	237.5 ± 49.8	<0.001
SU at Follow-up (μmol/L)	458.8 ± 40.6	330.5 ± 52.2	<0.001	392.9 ± 34.8	251.4 ± 49.6	<0.001
TG (mmol/L)	1.9 ± 1.3	1.5 ± 1.1	<0.001	1.6 ± 0.9	1.1 ± 0.7	<0.001
HDL-C (mmol/L)	1.2 ± 0.3	1.2 ± 0.3	<0.001	1.4 ± 0.3	1.5 ± 0.3	<0.001
TC (mmol/L)	4.9 ± 0.9	4.8 ± 0.9	<0.001	5.1 ± 1.0	4.8 ± 0.9	<0.001
LDL-C (mmol/L)	2.7 ± 0.8	2.7 ± 0.7	0.968	2.8 ± 0.8	2.6 ± 0.7	<0.001
FBG (mmol/L)	5.6 ± 1.3	5.7 ± 1.5	0.006	5.6 ± 1.3	5.2 ± 0.9	<0.001
BMI (Kg/m2)	25.8 ± 3.2	24.8 ± 3.2	<0.001	24.9 ± 3.5	22.7 ± 3.1	<0.001
WC (cm)	88.7 ± 8.9	86.4 ± 10.1	<0.001	80.5 ± 8.9	74.7 ± 8.1	<0.001
SBP (mmHg)	123.6 ± 14.3	123.0 ± 14.6	0.025	118.9 ± 16.1	112.2 ± 14.5	<0.001
DBP (mmHg)	81.6 ± 9.7	81.3 ± 9.6	0.043	76.8 ± 9.2	74.0 ± 8.9	<0.001

SU, serum urate; TG, total triglycerides; HDL-C, high-density lipoprotein cholesterol; TC, total cholesterol; LDL-C, low-density lipoprotein cholesterol; FBG, fasting blood glucose; BMI, body mass index; WC, waist circumference; SBP, systolic blood pressure; DBP, diastolic blood pressure; n, number.

^*^ Data were expressed as mean ± standard deviations. Apart from SU at Follow-up, which was measured at follow-up, the other variables were measured at baseline.

### Association between metabolism-related risk factors and SU

**Figure 1:**
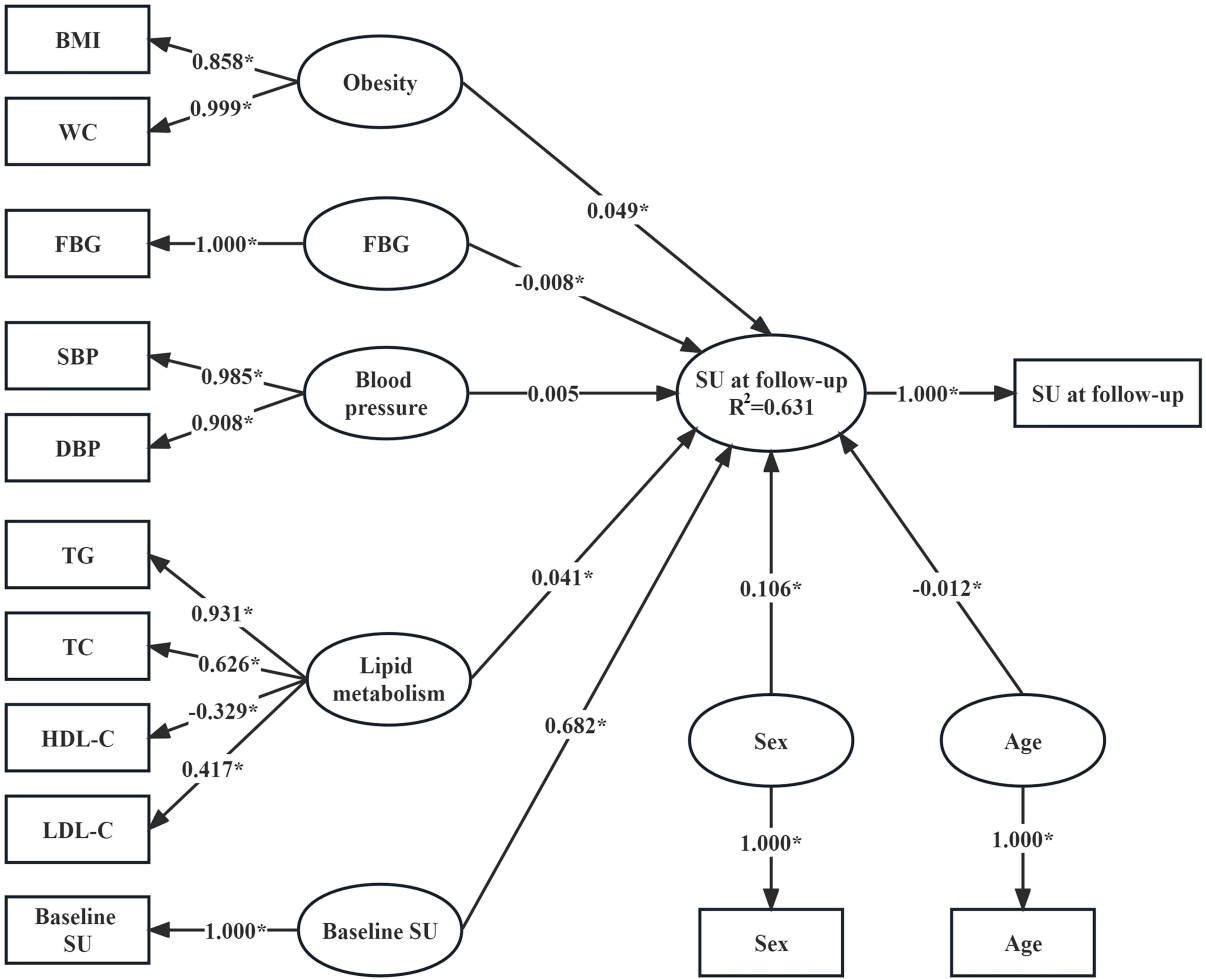
Association between SU concentrations at follow-up and baseline metabolism-related risk factors (n=48,979). Variables in the rectangle are observed variables, those in the ellipse are latent variables. Apart from SU at follow-up, which was measured at follow-up, the other observed variables were measured at baseline. The coefficients linking the latent variable “SU at follow-up” and other latent variables are the path coefficients (*β* coefficients) and they represent the direction and strength of the relations between the latent response variable “SU at follow-up” and other latent predictors. The coefficients linking the latent variables and the observed variables are the loadings (*λ* coefficients) and they represent in what direction and to what extent the observed variables reflect the latent variables. The R^2^ is the determination coefficient of the model where the latent “SU at follow-up” is the response variable and other latent variables are predictors, and it indicates the amount of variance in the latent response variable explained by its independent latent predictors. ^*^ *P*<0.05.

As shown in **Figure 1**, the determination coefficient (R^2^) of the PLSPM was 0.631, indicating that the model could explain 63.1% of the total variation. In the measurement model, WC had the largest contribution to obesity (loading = 0.999), followed by BMI (loading = 0.858). SBP (loading = 0.985) contributed slightly more to blood pressure than DBP (loading = 0.908). TG (loading = 0.931) had the largest contribution to lipid metabolism, followed by TC (loading = 0.626), again was LDL-C (loading = 0.417), and final was HDL-C (loading = -0.329). According to the structural model, baseline SU (coefficient = 0.682), lipid metabolism (coefficient = 0.041), sex (coefficient = 0.106) and obesity (coefficient = 0.049) were positively associated with SU at follow-up. Conversely, age (coefficient = -0.012) and FBG (coefficient = -0.008) were negatively associated with SU at follow-up.

### Subgroup and sensitivity analysis

**Supplementary Table S3** showed no significant difference in the estimation of the latent variables, the loading of the observed variables and the R^2^ of the models of different follow-up period groups. The results remain largely unchanged when using the cut-offs reported in the URRAH project to define hyperuricemia (**Supplementary Figure S2**).

### Associations of SU at follow-up with baseline FBG and age

Non-linear relationships were found between baseline FBG levels and SU at follow-up. A reversed U-shaped association was observed between baseline FBG concentration and SU at follow-up among men, with SU levels being highest when FBG concentration was approximately 6 mmol/L. In women, the SU at follow-up exhibited a positive relation with baseline FBG until the FBG concentration was approximately 7 mmol/L, then a phase of fluctuation of SU concentration was observed as FBG concentration progressed (**Supplementary Figure S3**). In addition, U-shaped relationships were found between baseline age and SU at follow-up, and the SU at follow-up was lowest when baseline age was approximately 57 years for men and 40 years for women (**Supplementary Figure S4**).

## Discussion

In this study, we observed that the incidence density of hyperuricemia was 78/1000 person-years. A previous study showed that the incidence density of hyperuricemia was 68.6/1000 person-years in China (15), and another Chinese study showed it was 43.6/1000 person-years (16). We noticed the incidence density in this study was higher than the previous results. This might be the result of increasing incidence of hyperuricemia due to the changes in lifestyles in China. Another reason could be the differences in the cut-off value used to define hyperuricemia.

In PLSPM models, we found that baseline SU is the most important factor associated with SU at follow-up. Previous study demonstrated that the risk of hyperuricemia is increased 2.33 times for every 1 mg/dL increase in baseline SU (17). Therefore, monitoring of SU concentration, identifying of high-risk individuals early and determining appropriate interventions timely will help to delay the occurrence or reduce the incidence of hyperuricemia.

In this study, we found that higher level of serum TG, TC, and LDL-C is directly associated with higher SU levels, whereas higher level of HDL-C is inversely associated with SU level, which is in agreement with the previous studies (18, 19). Thus, we proposed that lipid metabolism might have a regulatory role in urate homeostasis (20). In addition, men had a higher SU level compared to women in our research, which is consistent with previous studies (21, 22). This might be attributed to the innate metabolic characteristics and the acquired personal lifestyle (preference for alcohol, smoking, meat, etc. (23)) of men. In addition, our research showed that baseline BMI and WC, the predisposing risk factors of obesity, were positively associated with SU at follow-up. This result is consistent with previous studies that indicated obesity was one of the risk factors of hyperuricemia (1, 24).

This study revealed a non-linear trend between baseline FBG and SU at follow-up. In men, the results suggest an initial rise in SU levels with increasing FBG up to a critical cut-off, which was consistent with the observations reported in previous research (8, 25). However, above the cut-off value of FBG, SU concentration decreases with the increase of FBG level. The inverse relationship between SU and FBG above the cut-off value can be explained by increased glomerular filtration at higher FBG level, resulting in increased excretion of urate (8). In terms of renal tubular reabsorption of urate from glomerular filtrate, it was suggested that glucose at higher concentrations might compete with urate during urate reabsorption in the proximal tubule (26, 27) but that has not yet been experimentally verified. We also found that the SU concentration in women increased rapidly after 40 years, which might be related to menopause (28, 29). In postmenopausal women, a lower estrogen level causes increased urate reabsorption, resulting in higher SU levels (30). 

The strengths of the current study include a large sample size from the Han Chinese population in Beijing. Also, in this study we employed a relatively more updated approach called partial least squares path model (PLSPM; a structural equation model) to explore the interrelationship between SU and metabolism-related predisposing risk factors. This approach has its data-analytic capability and flexibility to include various important metabolism-related predisposing risk factors with a few restrictions on the distribution of variables (13). However, limitations should be noted. Personal lifestyle-related risk factors (e.g., smoking, drinking, physical activity, sedentary activity, sleep etc.) (31, 32) or drug usage (such as diuretics) (33) that may affect SU concentrations were not considered in this study due to data unavailable.

## Conclusions

This study revealed the associations of SU levels with sex, age and several metabolism-related risk factors, emphasizing the importance of these metabolism-related risk factors in SU management.

## Data availability statement

The authors are sure that all data and materials support the article. The datasets used in this study are available from the corresponding author upon reasonable request.

## Ethics statement

The study has been approved by Medical Ethics Committee of Beijing Physical Examination Center. The participants in this study have given informed consent and patient anonymity has been preserved.

## Author contributions

HL: Writing – review & editing. JZ: Formal analysis, Writing – original draft. HY: Formal analysis, Writing – review & editing. LD: Writing – review & editing. FL: Data curation, Formal analysis, Writing – review & editing. JW: Writing – review & editing.

## References

[1] Zhu Y Pandya BJ Choi HK. Prevalence of gout and hyperuricemia in the US general population: the National Health and Nutrition Examination Survey 2007-2008. Arthritis Rheum (2011) 63:3136–41. doi: 10.1002/art.30520 21800283

[2] Kumar AUA Browne LD Li X Adeeb F Perez-Ruiz F Fraser AD. Temporal trends in hyperuricaemia in the Irish health system from 2006-2014: A cohort study. PLoS One (2018) 13:e0198197. doi: 10.1371/journal.pone.0198197 29852506 PMC5980488

[3] Maloberti A Qualliu E Occhi L Sun J Grasso E Tognola C. Hyperuricemia prevalence in healthy subjects and its relationship with cardiovascular target organ damage. Nutr Metab Cardiovasc Dis (2021) 31:178–85. doi: 10.1016/j.numecd.2020.08.015 32994122

[4] Maloberti A Rebora P Andreano A Vallerio P De Chiara B Signorini S. Pulse wave velocity progression over a medium-term follow-up in hypertensives: Focus on uric acid. J Clin Hypertens (Greenwich) (2019) 21:975–83. doi: 10.1111/jch.13603 PMC8030372 31222917

[5] Redon P Maloberti A Facchetti R Redon J Lurbe E Bombelli M. Gender-related differences in serum uric acid in treated hypertensive patients from central and east European countries: findings from the Blood Pressure control rate and CArdiovascular Risk profilE study. J Hypertens (2019) 37:380–8. doi: 10.1097/HJH.0000000000001908 30074564

[6] Lohsoonthorn V Dhanamun B Williams MA. Prevalence of hyperuricemia and its relationship with metabolic syndrome in Thai adults receiving annual health exams. Arch Med Res (2006) 37:883–9. doi: 10.1016/j.arcmed.2006.03.008 16971230

[7] Liu R Han C Wu D Xia XH Gu JQ Guan HX. Prevalence of hyperuricemia and gout in Mainland China from 2000 to 2014: A systematic review and meta-analysis. BioMed Res Int (2015) 2015:762820. doi: 10.1155/2015/762820 26640795 PMC4657091

[8] Li Q Yang Z Lu B Wen J Ye Z Chen LL. Serum uric acid level and its association with metabolic syndrome and carotid atherosclerosis in patients with type 2 diabetes. Cardiovasc Diabetol (2011) 10:72. doi: 10.1186/1475-2840-10-72 21816063 PMC3163178

[9] Liu DM Jiang LD Gan L Su Y Li F. Association between serum uric acid level and body mass index in sex- and age-specific groups in southwestern China. Endocr Pract (2019) 25:438–45. doi: 10.4158/EP-2018-0426 30657365

[10] Duan Z Fu J Zhang FF Cai Y Wu G Ma W. The association between BMI and serum uric acid is partially mediated by gut microbiota. Microbiol Spectr (2023) 11:e0114023. doi: 10.1128/spectrum.01140-23 37747198 PMC10581133

[11] Multi-disciplinary expert task force on hyperuricemia and its related diseases Zou HJ Wu HS Zhou JG Zeng XJ Dai L. Chinese multi-disciplinary consensus on the diagnosis and treatment of hyperuricemia and its related diseases. Chin J Intern Med (2017) 56:235–48. doi: 10.3760/cma.j.issn.0578-1426.2017.03.021 28253612

[12] Maloberti A Giannattasio C Bombelli M Desideri G Cicero AFG Muiesan ML. Hyperuricemia and risk of cardiovascular outcomes: the experience of the URRAH (Uric acid right for heart health) project. High Blood Press Cardiovasc Prev (2020) 27(2):121–8. doi: 10.1007/s40292-020-00368-z 32157643

[13] Tenenhaus M Vinzi VE Chatelinc YM Laurob C. PLS path modeling. Comput Stat Data Anal (2005) 48:159–205. doi: 10.1016/j.csda.2004.03.005

[14] Sanchez G. PLS Path Modeling with R. Berkeley (2013). Available at: http://www.gastonsanchez.com/PLS.

[15] Ni Q Lu X Chen C Du H Zhang R. Risk factors for the development of hyperuricemia: A STROBE-compliant cross-sectional and longitudinal study. Med (Baltimore) (2019) 98:e17597. doi: 10.1097/MD.0000000000017597 PMC6824661 31626136

[16] Zeng C Wei J Yang T Li H Xiao WF Luo W. Higher blood hematocrit predicts hyperuricemia: a prospective study of 62,897 person-years of follow-up. Sci Rep (2015) 5:13765. doi: 10.1038/srep13765 26337238 PMC4559718

[17] McAdams-DeMarco MA Law A Maynard JW Coresh J Baer AN. Risk factors for incident hyperuricemia during mid-adulthood in African American and white men and women enrolled in the ARIC cohort study. BMC Musculoskelet Disord (2013) 14:347. doi: 10.1186/1471-2474-14-347 24330409 PMC3878839

[18] Xu J Peng H Ma QH Zhou XH Xu WX Huang L. Associations of non-high density lipoprotein cholesterol and traditional blood lipid profiles with hyperuricemia among middle-aged and elderly Chinese people: a community-based cross-sectional study. Lipids Health Dis (2014) 13:117. doi: 10.1186/1476-511X-13-117 25052552 PMC4115491

[19] Peng TC Wang CC Kao TW Chan JY Yang YH Chang YW. Relationship between hyperuricemia and lipid profiles in US adults. BioMed Res Int (2015) 2015:127596. doi: 10.1155/2015/127596 25629033 PMC4299312

[20] Maloberti A Vanoli J Finotto A Bombelli M Facchetti R Redon P. Uric acid relationships with lipid profile and adiposity indices: Impact of different hyperuricemic thresholds. J Clin Hypertens (Greenwich) (2023) 25:78–85. doi: 10.1111/jch.14613 36573350 PMC9832232

[21] Zitt E Fischer A Lhotta K Concin H Nagel G. Sex- and age-specific variations, temporal trends and metabolic determinants of serum uric acid concentrations in a large population-based Austrian cohort. Sci Rep (2020) 10:7578. doi: 10.1038/s41598-020-64587-z 32371883 PMC7200724

[22] Kim Y Kang J Kim GT. Prevalence of hyperuricemia and its associated factors in the general Korean population: an analysis of a population-based nationally representative sample. Clin Rheumatol (2018) 37:2529–38. doi: 10.1007/s10067-018-4130-2 29790110

[23] Yu SS Yang HM Guo XF Zhang XG Zhou Y Ou QY. Prevalence of hyperuricemia and its correlates in rural Northeast Chinese population: from lifestyle risk factors to metabolic comorbidities. Clin Rheumatol (2016) 35:1207–15. doi: 10.1007/s10067-015-3051-6 26292632

[24] Trifirò G Morabito P Cavagna L Ferrajolo C Pecchioli S Simonetti M. Epidemiology of gout and hyperuricaemia in Italy during the years 2005-2009: a nationwide population-based study. Ann Rheum Dis (2013) 72:694–700. doi: 10.1136/annrheumdis-2011-201254 22736095

[25] Cheserek MJ Shi Y Le G. Association of hyperuricemia with metabolic syndrome among university workers: sex and occupational differences. Afr Health Sci (2018) 18:842–51. doi: 10.4314/ahs.v18i4.2 PMC6354883 30766547

[26] Tuomilehto J Zimmet P Wolf E Taylor R Ram P King H. Plasma uric acid level and its association with diabetes mellitus and some biologic parameters in a biracial population of Fiji. Am J Epidemiol (1988) 127:321–36. doi: 10.1093/oxfordjournals.aje.a114807 3337086

[27] Herman JB Medalie JH Goldbourt U. Diabetes, prediabetes and uricaemia. Diabetologia (1976) 12:47–52. doi: 10.1007/BF01221964 1254115

[28] Hak AE Choi HK. Menopause, postmenopausal hormone use and serum uric acid levels in US women–the Third National Health and Nutrition Examination Survey. Arthritis Res Ther (2008) 10:R116. doi: 10.1186/ar2519 18822120 PMC2592803

[29] Lin KC Lin HY Chou P. Community based epidemiological study on hyperuricemia and gout in Kin-Hu, Kinmen. J Rheumatol (2000) 27:1045–50. 10782835

[30] Koga M Saito H Mukai M Kasayama S Yamamoto T. Factors contributing to increased serum urate in postmenopausal Japanese females. Climacteric (2009) 12:146–52. doi: 10.1080/13697130802607719 19105055

[31] Ryu S Chang Y Zhang Y Kim SG Cho J Son HJ. A cohort study of hyperuricemia in middle-aged South Korean men. Am J Epidemiol (2012) 175:133–43. doi: 10.1093/aje/kwr291 22156041

[32] Park DY Kim YS Ryu SH Jin YS. The association between sedentary behavior, physical activity and hyperuricemia. Vasc Health Risk Manag (2019) 15:291–9. doi: 10.2147/VHRM.S200278 PMC6698593 31616149

[33] Maloberti A Bombelli M Facchetti R Barbagallo CM Bernardino B Rosei EA. Relationships between diuretic-related hyperuricemia and cardiovascular events: data from the URic acid Right for heArt Health study. J Hypertens (2021) 39:333–40. doi: 10.1097/HJH.0000000000002600 33239553

