# Supplementary material for: Incidence density of hyperuricemia and association between metabolism-related predisposing risk factors and serum urate in Chinese adults: a cohort study
Source: Front Endocrinol (Lausanne). 2023 Dec 7;14:1253470. doi: 10.3389/fendo.2023.1253470 (PMC10733531; doi:10.3389/fendo.2023.1253470)
Supplement: Supplementary file 1 [file DataSheet_1.docx]

**Supplementary Material**

**Supplementary Table S1** Incidence density of hyperuricemia in participants ≥18 years in Beijing Physical Examination Center

**Supplementary Table S2** Prevalence of hyperuricemia in participants ≥18 years in Beijing Physical Examination Center

**Supplementary Table S3** Parameters of the PLSPM in the population grouped by follow-up time

**Supplementary Fig. S1** Flow chart of the study

**Supplementary Fig. S2** Association between SU concentrations at follow-up and baseline metabolism-related risk factors when using the cut-offs founded in the URRAH project (>5.1 mg/dL for females and >5.6 mg/dL for males) to define hyperuricemia (n=33,130)

**Supplementary Fig. S3** The sex-specific relationships between SU concentrations at-follow and baseline FBG concentrations

**Supplementary Fig. S4** The sex-specific relationships between SU concentrations at-follow and baseline age

**Supplementary Table S1** Incidence density of hyperuricemia in participants ≥18 years in Beijing Physical Examination Center

| Variable | Number of new cases of hyperuricemia | Person time (person-years) | Incidence density (1/1000 person-years) |
| --- | --- | --- | --- |
| Men | 4,259 | 37,086 | 115 |
| Women | 1,560 | 37,288 | 42 |
| Total | 5,819 | 74,374 | 78 |

**Supplementary Table S2** Prevalence of hyperuricemia in participants ≥18 years in Beijing Physical Examination Center

| Year | Number of cases of hyperuricemia | Number of the total population | Prevalence (%) |
| --- | --- | --- | --- |
| 2014 | 10,983 | 71,964 | 15 |
| 2015 | 12,260 | 70,983 | 17 |
| 2016 | 12,659 | 66,221 | 19 |

**Supplementary Table S3** Parameters of the PLSPM in the population grouped by follow-up time

| Subgroup | Latent variable | Path coefficients | Observed variable | Loading | R^2^ |
| --- | --- | --- | --- | --- | --- |
| 1 | SU | - | SU | 1.000^*^ | 0.656 |
|  | Age | -0.011 | Age | 1.000^*^ |  |
|  | Sex | 0.106^*^ | Sex | 1.000^*^ |  |
|  | FBG | -0.003 | FBG | 1.000^*^ |  |
|  | Baseline SU | 0.673^*^ | Baseline SU | 1.000^*^ |  |
|  | Obesity | 0.071^*^ | BMI | 0.894^*^ |  |
|  |  |  | WC | 0.999^*^ |  |
|  | Lipid metabolism | 0.048^*^ | TG | 0.924^*^ |  |
|  |  |  | TC | 0.660^*^ |  |
|  |  |  | HDL-C | -0.323^*^ |  |
|  |  |  | LDL-C | 0.442^*^ |  |
|  | Blood pressure | 0.015^*^ | SBP | 0.985^*^ |  |
|  |  |  | DBP | 0.913^*^ |  |
| 2 | SU | - | SU | 1.000^*^ | 0.619 |
|  | Age | -0.005 | Age | 1.000^*^ |  |
|  | Sex | 0.111^*^ | Sex | 1.000^*^ |  |
|  | FBG | -0.006 | FBG | 1.000^*^ |  |
|  | Baseline SU | 0.677^*^ | Baseline SU | 1.000^*^ |  |
|  | Obesity | 0.047^*^ | BMI | 0.829^*^ |  |
|  |  |  | WC | 0.999^*^ |  |
|  | Lipid metabolism | 0.040^*^ | TG | 0.929^*^ |  |
|  |  |  | TC | 0.619^*^ |  |
|  |  |  | HDL-C | -0.323^*^ |  |
|  |  |  | LDL-C | 0.421^*^ |  |
|  | Blood pressure | -0.004 | SBP | 0.986^*^ |  |
|  |  |  | DBP | 0.901^*^ |  |
| 3 | SU | - | SU | 1.000^*^ | 0.628 |
|  | Age | -0.021^*^ | Age | 1.000^*^ |  |
|  | Sex | 0.106^*^ | Sex | 1.000^*^ |  |
|  | FBG | -0.024^*^ | FBG | 1.000^*^ |  |
|  | Baseline SU | 0.694^*^ | Baseline SU | 1.000^*^ |  |
|  | Obesity | 0.031^*^ | BMI | 0.886^*^ |  |
|  |  |  | WC | 0.999^*^ |  |
|  | Lipid metabolism | 0.032^*^ | TG | 0.939^*^ |  |
|  |  |  | TC | 0.609^*^ |  |
|  |  |  | HDL-C | -0.352^*^ |  |
|  |  |  | LDL-C | 0.397^*^ |  |
|  | Blood pressure | 0.011 | SBP | 0.984^*^ |  |
|  |  |  | DBP | 0.917^*^ |  |

SU: serum urate; TG: total triglycerides; HDL-C: high-density lipoprotein cholesterol; TC: total cholesterol; LDL-C: low-density lipoprotein cholesterol; FBG: fasting blood glucose; BMI: body mass index; WC: waist circumference; SBP: systolic blood pressure; DBP: diastolic blood pressure.

Subgroup 1 (number=12470): the participants with follow-up time ≤1 year; Subgroup 2 (number =24907): the participants with follow-up time >1 year and ≤2 years; Subgroup 3 (number =11602): the participants with follow-up time >2 years and ≤3 years

^*^*p* <0.05


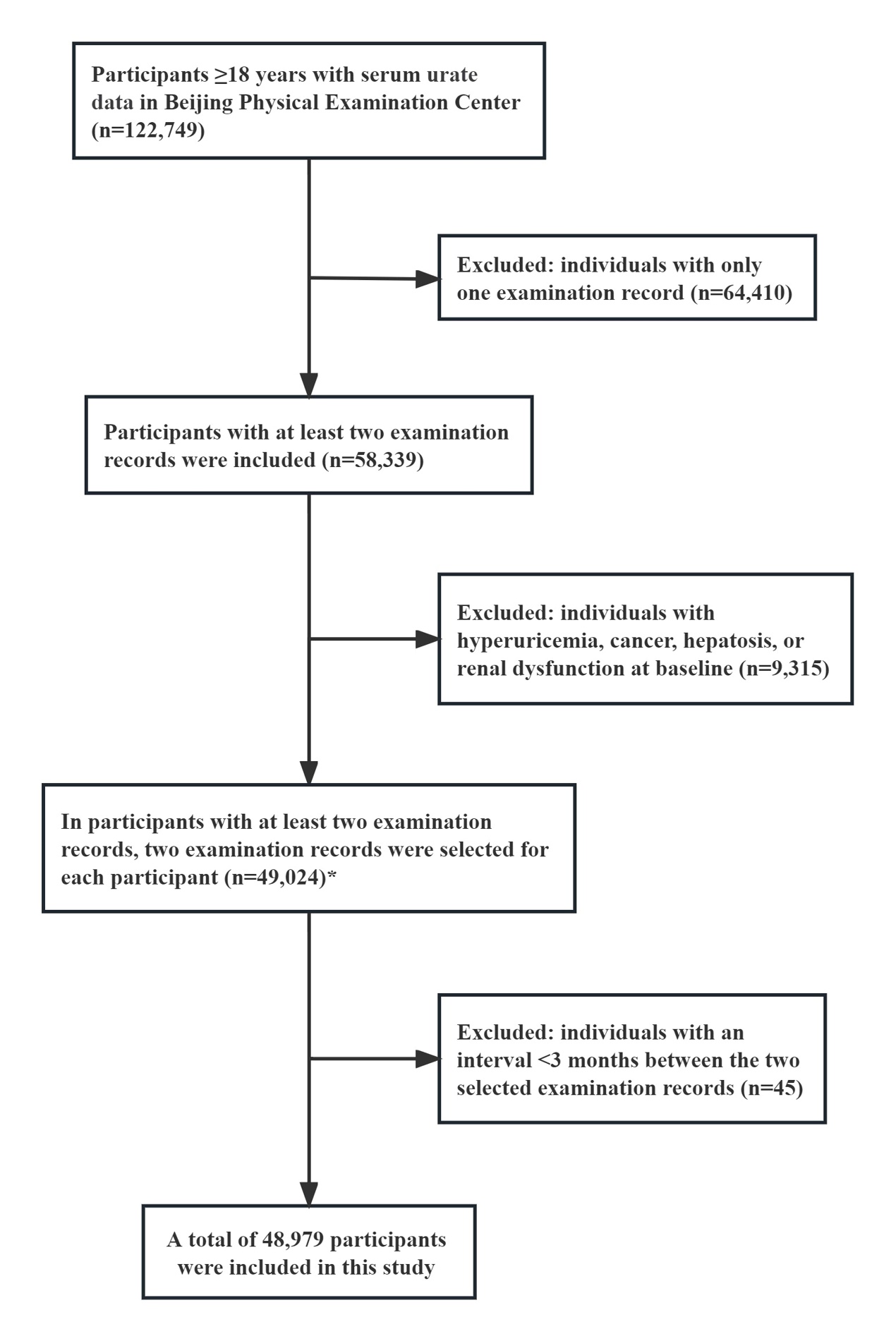


**Supplementary Fig. S1** Flow chart of the study

**^*^**The selection criteria for two examination records for each participant are as follows: 1. Participants with only two examination records have both records included. 2. For participants with more than two examination records: a) If hyperuricemia is absent in all examination records, then the first and last examination records are chosen. b) If hyperuricemia is diagnosed in any of the follow-up examination records, then the first examination record and the earliest one with hyperuricemia diagnosis are selected.


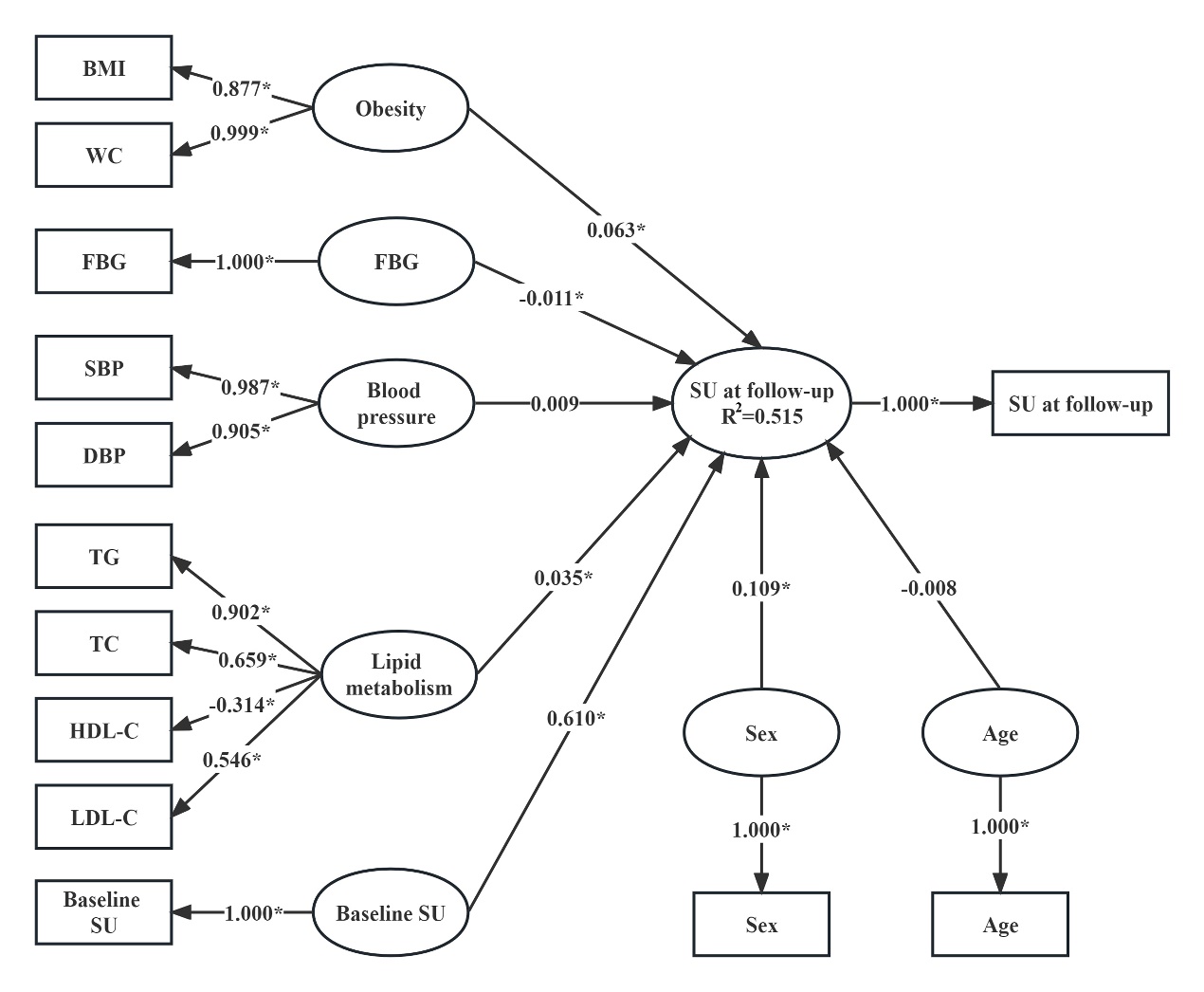


**Supplementary Fig. S2** Association between SU concentrations at follow-up and baseline metabolism-related risk factors when using the cut-offs founded in the URRAH project (>5.1 mg/dL for females and >5.6 mg/dL for males) to define hyperuricemia (n=33,130)**^†^**

**^†^**Variables in the rectangle are observed variables, those in the ellipse are latent variables. Apart from SU at Follow-up, which was measured at follow-up, the other observed variables were measured at baseline. The coefficients between the latent variable “SU at follow-up” and other latent variables are the path coefficients (*β* coefficients) and they represent the direction and strength of the relations between the latent response variable “SU at follow-up” and other latent predictors. The coefficients between the latent variables and the observed variables are the loadings (*λ* coefficients) and they represent in what direction and to what extent the observed variables reflect the latent variables. The R^2^ is the determination coefficient of the model where the latent "SU at follow-up" is the response variable and other latent variables are predictors, and it indicates the amount of variance in the latent response variable explained by its independent latent predictors.

^*^*p* <0.05


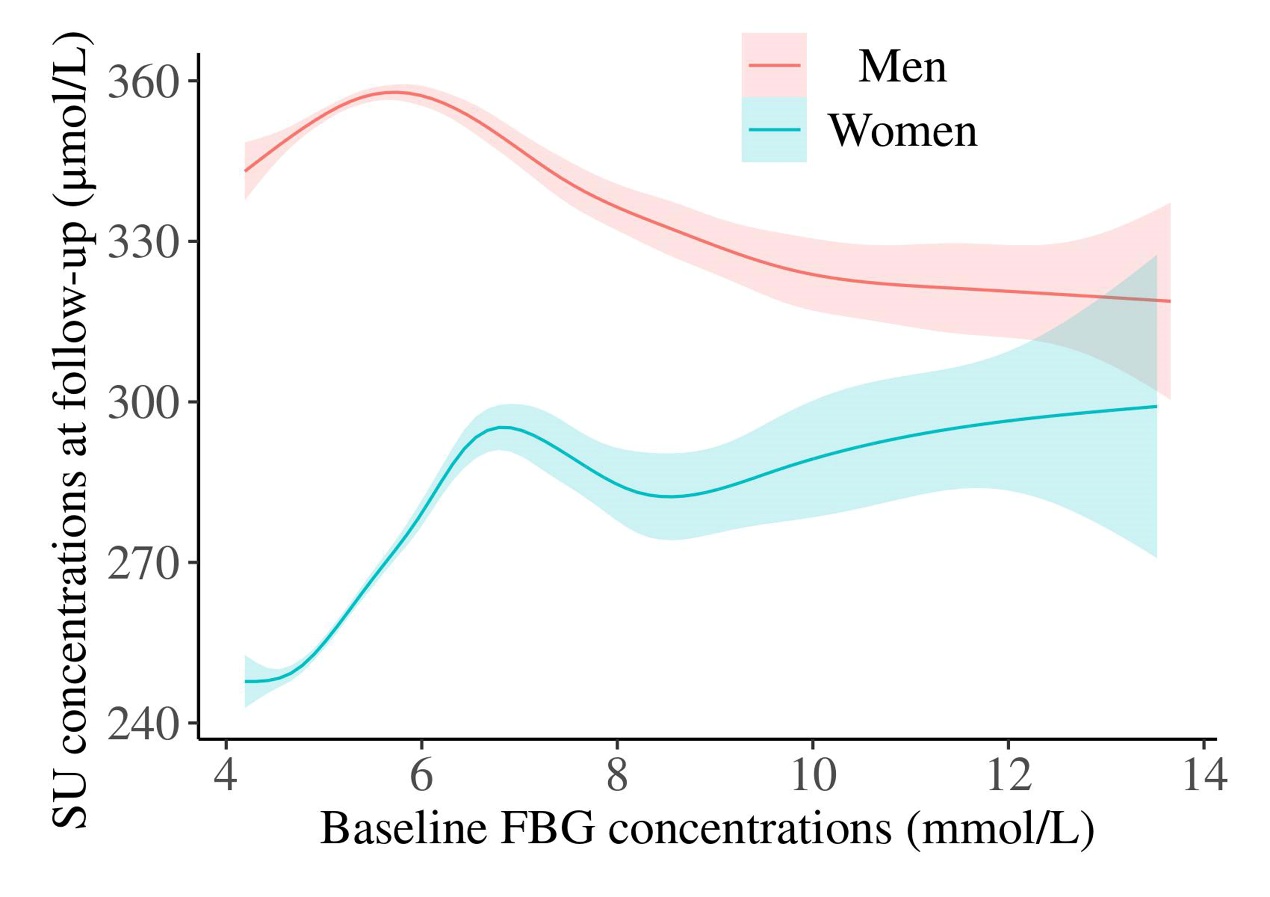


**Supplementary Fig. S3** The sex-specific relationships between SU concentrations at-follow and baseline FBG concentrations. The solid lines represent the point estimates and the shaded areas indicate 95% confidence intervals.


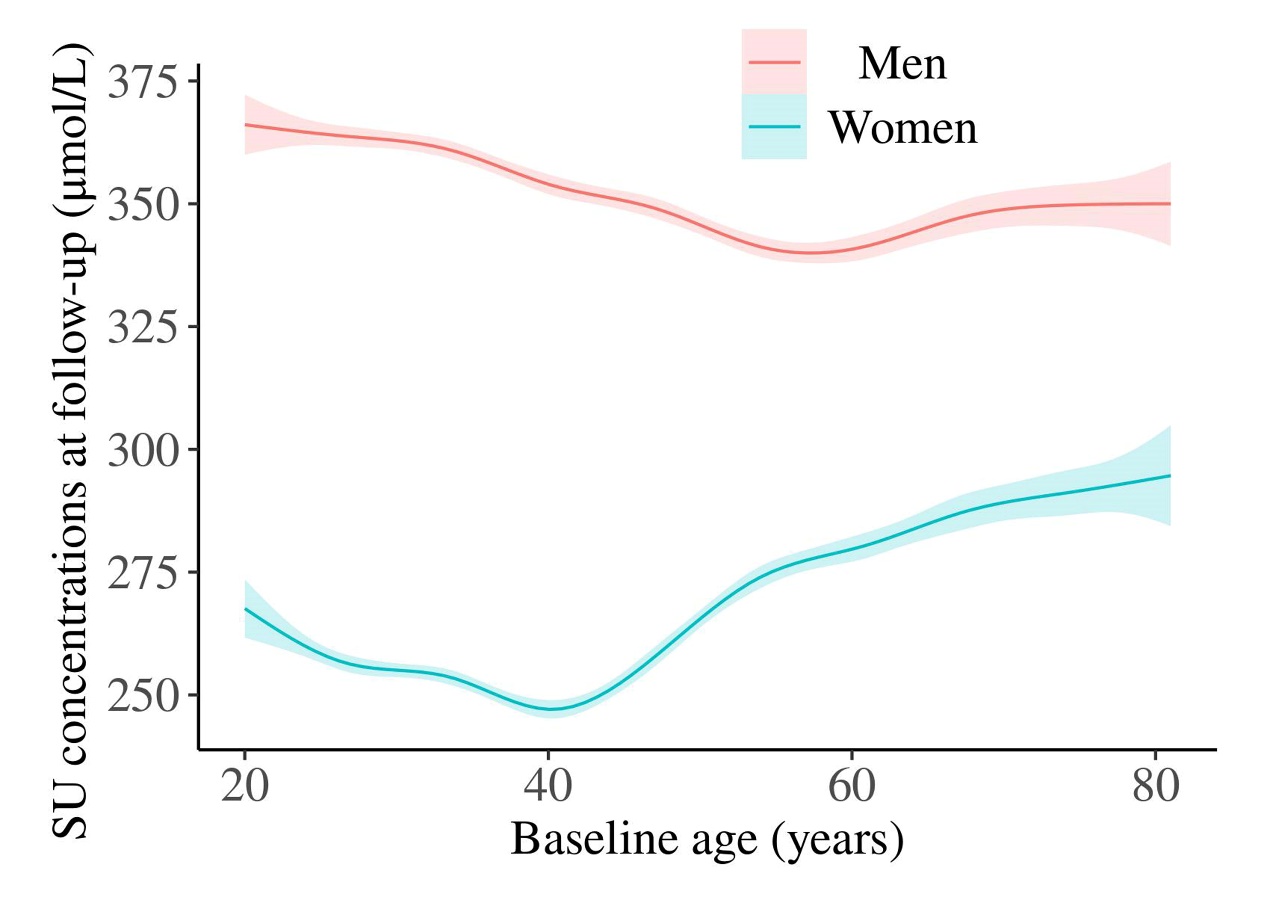


**Supplementary Fig. S4** The sex-specific relationships between SU concentrations at-follow and baseline age. The solid lines represent the point estimates and the shaded areas indicate 95% confidence intervals.
